# The UroLift implant: mechanism behind rapid and durable relief from prostatic obstruction

**DOI:** 10.1038/s41391-021-00434-0

**Published:** 2021-08-06

**Authors:** Claus G. Roehrborn, Peter T. Chin, Henry H. Woo

**Affiliations:** 1 The University of Texas Southwestern Medical Center, Dallas, TX USA; 2 School of Medicine, University of Wollongong & SouthCoast Urology, Wollongong, NSW Australia; 3 College of Health and Medicine, Australian National University & The University of Sydney & SAN Prostate Centre of Excellence, Sydney Adventist Hospital, Wahroonga, NSW Australia

**Keywords:** Prostatic diseases, Translational research

## Abstract

**Background:**

Benign prostatic hyperplasia (BPH) is an affliction of the aging male population that contributes to bothersome and disruptive lower urinary tract symptoms (LUTS). The UroLift® implant has been developed as a mechanical means of widening the prostatic urethra and providing relief from lower urinary tract symptoms (LUTS) through a minimally invasive procedure.

**Methods:**

In the current study, we utilize histological results from canine tissue, resected tissue from human subjects treated with the UroLift System and post-market surveillance data collected by the manufacturer in order to elucidate the long-term biological mechanism of action of the UroLift implant.

**Results:**

The delivery of the implant causes tissue compression, likely resulting in focal ischemia that causes observed local atrophy and minimal-mild chronic inflammation that ultimately remodels tissue to produce a widened prostatic urethra.

**Conclusions:**

These studies reveal the lack of impact the device has on systemic tissue, providing evidence that the UroLift System is benign and biocompatible, and offering histologic explanation for the clinically observed durability.

## Introduction

Implantable medical devices are widely used in the therapeutic management of a broad range of medical conditions. It is estimated that approximately 8–10% of the US population and 5–6% in industrialized countries have been implanted with some type of a medical device to treat an existing medical condition, restore function, improve the quality of life, and in many cases, extend patient longevity [1]. Permanent implants have not only represented tremendous advancement for how certain disease are treated but have also changed the standard of care by providing patients with a minimally invasive alternative option to traditional surgery. For example, in the US, nearly four million artificial intra-ocular lenses [2], 8000 cochlear implants [3], and 16,000 stent grafts for abdominal aortic aneurysms are implanted into patients each year [4]. These devices serve to reduce risks of mortality, improve quality of life by returning sight and hearing, and avoid the risks of open cavity surgery.

Within the field of interventional urology, a standard technique for nerve sparing radical prostatectomy is to refrain from cautery and instead deploy ligation clips to arterial bleeds at the level of the prostate [5]. The use of small permanent implants, in this case, reduces the likelihood of thermal damage to the neurovascular bundles that could induce erectile dysfunction. Perhaps paradoxically, the application of large amounts of heat to the prostate has been the surgical standard for the treatment of benign prostatic hyperplasia for nearly 100 years. In 1980, Fabian reported the use of the first metallic intraprostatic stent in an attempt to open the occluded prostate without surgery or heat [6]. Over the next decade, several metal mesh and spiral stents were developed for the prostatic urethra. Although initially successful at improving LUTS, up to 47% of implanted men required removal of their prostatic stents due to complications associated with migration and encrustation, with most removals occurring within two years of the initial procedure [7]. Despite later permutations aimed at overcoming these limitations, the use of prostatic stents was largely abandoned due to high rates of migration, encrustation, and device failure [8].

The Prostatic Urethral Lift (PUL) procedure utilizing the UroLift® System is a very different approach to mechanically opening the prostatic urethra that minimizes permanent implant material and its exposure to the urinary system. Rather than placing a tube-like structure within the urethra like the permanent stents introduced in the 1980s, the UroLift implants (Teleflex, Pleasanton, CA) are deployed transprostatically such that only a small metal tab rests on the urethra, and deployment tension causes that tab and the urethral wall under it to invaginate into the adenoma. Approved by the US FDA in 2013, the UroLift System is the first mechanical prostatic implant to demonstrate safe and effective treatment of BPH without the clinical sequelae of prior stents, such as a migration, and high rates of encrustation, infection, and need for removal if deployed properly. The safety profile shows mild to moderate side effects that largely resolve by two to four weeks post treatment [9]. Durability in symptom improvement has been demonstrated through five years with a surgical retreatment rate of 13.6% [10]. Although extensive evidence has established the clinical outcomes of PUL with the UroLift System [10–14], little has been published regarding the short-term and long-term mechanism of action of the implants. Here we analyze pre-clinical in vivo and in vitro test results, samples and images from clinical trial subjects, and post-market surveillance data collected by the manufacturer in order to elucidate the long-term biological mechanism of action and assess the likelihood of implant migration, encrustation, and breakage that underly the established treatment durability.

## Methods

### Prostatic urethral lift procedure

**Fig. 1:**
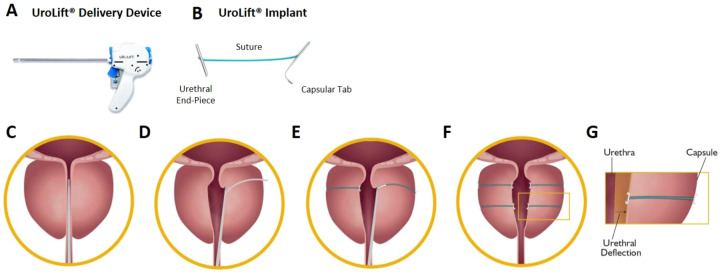
UroLift System Components and Procedure. The UroLift System is comprised of two main components: (**A**) the UroLift Delivery Device and (**B**) the UroLift implant. The delivery device is designed to access the prostatic urethra and deliver one implant through the lobes of the prostate. The implant consists of a Capsular Tab (CT), made from nitinol (nickel titanium alloy), connected by a monofilament polyethylene terephthalate suture to the Urethral End-Piece (UE), made from stainless steel. The materials used in the implant are made of chemically and biologically inactive materials, commonly used in other implants. Details of the PUL procedure are as follows: (**C**) under cystoscopic guidance the delivery device is introduced through the sheath and is used to compress the prostate lobe. **D** A 19-gauge needle that houses the implant is deployed through the prostatic lobe and capsule. Upon retraction of the needle, the CT is deposited with a suture under tension. **E** The implant is secured by deployment of the UE and excess suture is cut. (**F**) Additional implants are delivered as required. **G** The glandular stromal tissue of the prostate is compliant and more easily compressed outwardly resulting in the opening of the prostatic urethra.

The Prostatic Urethral Lift procedure involves the cystoscopically guided transurethral deployment of small permanent UroLift implants transversely across the lobes of the prostate. Rigid cystoscopy is performed, and a delivery device is introduced through the sheath. The device is used to compress the prostate lobe at the desired location, and a 19-gauge hollow needle that houses the implant is deployed into the prostate such that it passes through the prostatic capsule. Upon retraction, the needle deposits a nitinol tab on the capsular surface and a tensioned suture from the tab to the device. A stainless steel endpiece is then affixed to the tensioned suture at the urethral surface. The suture is then cut, completing the deployment (Fig. 1). Because the glandular stromal tissue of the prostate is compliant and more easily compressed outwardly than the prostatic capsule pulled inwardly, compression results in the opening of the prostatic urethra (Fig. 1G). Typically, four to six implants are required per treatment to open the anterior aspect of the prostatic urethra from bladder neck to prostatic apex, thus reducing the prostatic obstruction.

### Biocompatibility testing and animal tissue studies

To evaluate the chronic tissue response to the UroLift implant, pre-clinical studies were carried out in dogs following GLP quality systems. A total of 24 healthy dogs were implanted with the UroLift device. Sham (control) group included 15 animals that underwent the same procedure, including deployment of the needle, except for the placement of the implant. Following the intervention, operative success, post-op bleeding, and injury were evaluated. Follow-up cystoscopy and fluoroscopy examinations were performed just prior to sacrifice at 1 (*n* = 8), 3 (*n* = 3), 6 (*n* = 8), and 12 months (*n* = 5) after implantation. The removed specimens were then subjected to gross examination and histopathological studies.

### Human tissue studies

Tissue specimens containing components of the UroLift implant with the surrounding urethra and prostate tissue were collected from four patients enrolled and treated in an early PUL feasibility clinical trial (ACTRN12609000760279) who underwent transurethral resection of the prostate at 13, 15, 27, and 43 months after PUL treatment. Histological studies were performed on the excised tissue.

### Histopathology

All tissue samples, human and canine, were fixed in 10% formalin, embedded in Spurr’s resin, cut into 4 μm sections, and stained with hematoxylin and eosin (H&E). Microscopic evaluation was focused on any potential changes attributed to the implant including tissue damage, thrombosis, encrustation, inflammation, abnormal fibrosis, infection, and proliferative changes, as well as wound healing processes in tissues surrounding the implant. Measurements were conducted using ImageJ.

### Scanning electron microscope inspection

To evaluate the long-term integrity of the Urolift implant, three Urolift explants were inspected with scanning electron microscopy (SEM) after 15 months of implantation in canine prostate. Explant surfaces were examined for any visible sign of corrosion or degradation, and surface properties were compared to an unused implant control. The SEM inspection was performed using a FEI Quanta 200 3D microscope operated at 20 kV in the secondary electron mode at magnification ranging from 39X to 500X. Both the Inside Diameter (ID) and Outside Diameter (OD) were evaluated during the inspection.

### Post-market data analysis

Worldwide post-market data spanning April 2018 through September 2020 were made available by the implant manufacturer (Teleflex, Pleasanton, CA) and reviewed for cases of implant migration, encrustation, and breakage. Commercial complaints reporting was utilized to deduce the migration, breakage and stone formation rates. The search terms utilized for potential breakage incidents were, “Dislodged urethral endpiece, dislodged implant, unattached implant, loose urethral endpiece, and poorly tensioned implant.” For potential stone formation incidents, the search terms included “Stone formation, urinary calculus, and encrustation/stone formation.”

## Results

### Evidence of acute mechanical mode of action

**Fig. 2:**
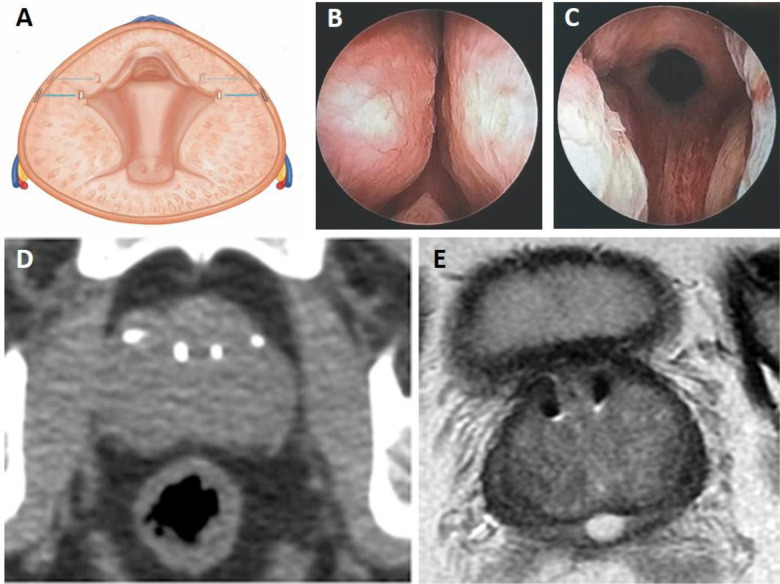
Visualization of UroLift® Implant Placement. **A** Illustration of UroLift implants placed as a result of the PUL procedure, which reside in the anterolateral prostate and anterior to the neurovascular bundles. Cystoscopy images captured (**B**) before and (**C**) after PUL demonstrating a continuous channel through the anterior aspect of the prostatic urethra. **D** Computed tomography (CT) and (**E**) MRI scans depicting the final implant location following treatment.

**Fig. 3:**
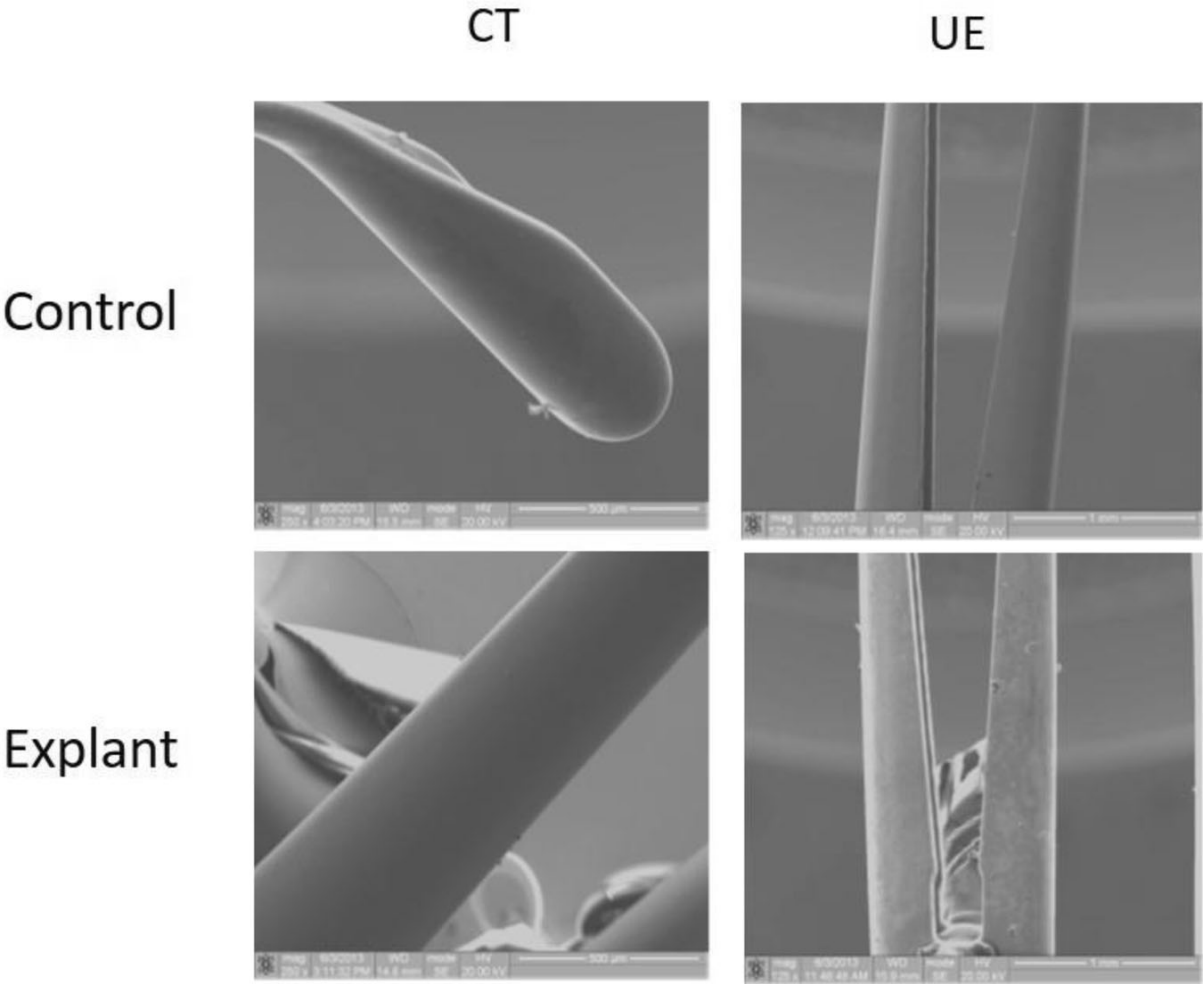
Durability of the UroLift® Implant. SEM images of surfaces of Capsular Tab (CT), and Urethral End-piece (UE) of a Urolift explant after 15 months of implantation in canine prostate, compared to unused control sample. Note the similarity between the explant surface and that of control, with no visible signs of damage or corrosion on the explant.

PUL is intended to reduce obstruction specifically in the anterior aspect of the prostatic urethra (Fig. 2a). Evidence supporting this mechanism are: (1) cystoscopy at end of procedure (Fig. 2c), (2) computed tomography (CT) scan of prostate (Fig. 2d), and (3) magnetic resonance imaging (MRI) of the prostate (Fig. 2e), as well as the symptomatology of patients over time. Each imaging technique demonstrates the increased opening of the prostatic urethra thereby reducing prostatic urethral obstruction. CT images show location of both urethral and capsular components, whereas the nitinol capsular tab is not visible on MRI. A small artifact surrounds the MR image of the urethral end piece in T2 weighted imaging. As evidenced in the clinical studies of PUL, obstructive or voiding measures of the International Prostate Symptom Score (IPSS) is significantly reduced by two weeks, and irritative or storage symptoms follow thereafter reaching peak response by three months [15]. Urinary flow rates improve without reaching levels that are associate with cavitating procedures where most or all prostate glandular tissue is removed. Recent publications show a high success rate in treating men in acute and chronic urinary retention, freeing them from daily urinary catheterization [12, 16].

### Biocompatibility & histology studies

Histopathological evaluation of the implant and surrounding tissue was carried out by an independent pathologist using their standardized grading system of tissue response. extracted from canine specimens at 1, 3, 6, and 12 months post implantation revealed no signs of hemorrhage, infection, necrosis, encrustation, and exaggerated tissue proliferation. Microscopic examination of the implant-tissue interface (where the implant is in direct contact with surrounding tissue) showed minimal-mild chronic inflammation at one-month post implantation, and minimal chronic inflammation and minimal-mild fibrosis at six and 12 months post implantation, which are signs of a stable and normal healing response typical of a permanent biocompatible device [17, 18]. Proliferative changes indicative of aberrant tissue response such as urothelial or glandular hyperplasia or neoplasia were not observed at any timepoint evaluated. Inspection of the implant surface with SEM performed on three UroLift explants after 15 months of implantation in canines demonstrated no signs of corrosion or degradation when compared to an unused control (Fig. 3).

**Fig. 4:**
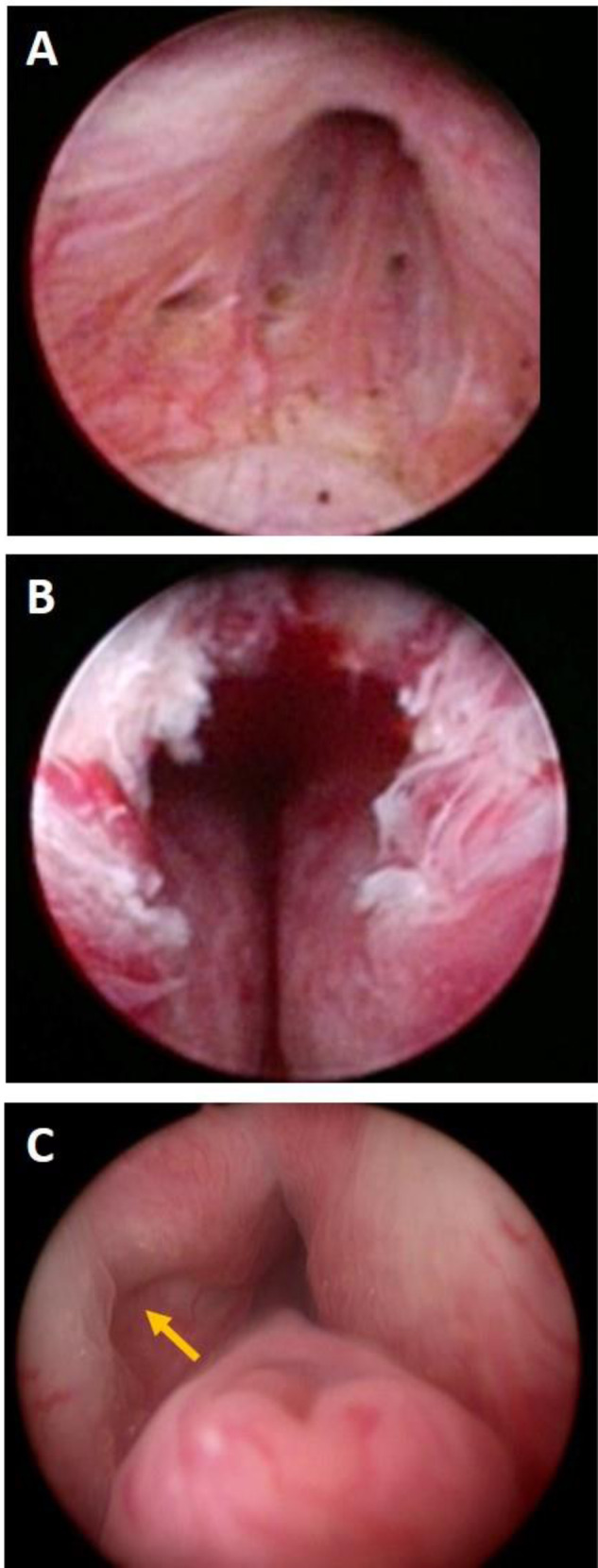
Inflammatory response and invagination of the UroLift implant following treatment. **A** Cystoscopic images of apex at baseline, (**B**) at 12 months post treatment and (**C**) at 27 months post treatment show absence of inflammation at 12 and 27 months post implantation. Arrow indicates invaginated tissue where an implant had been placed. The urethra in that area shows no difference from the healthy urethra on the contralateral side.

**Fig. 5:**
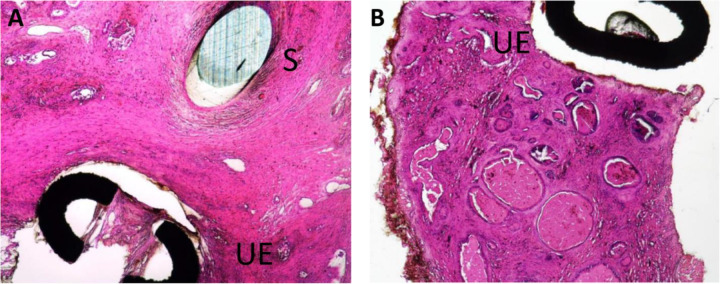
Atrophic response following UroLift implant placement. **A** Mild fibrosis or encapsulation at the tissue interface of the Urethral End-Piece (UE) and Suture (S) component of UroLift implant removed from a patient who underwent transurethral resection at 43 months post-implantation of the device. **B** No abnormalities at the tissue interface of the Urethral End-Piece and mild multifocal glandular dilation in the surrounding parenchyma.

Available human data corroborate the findings of the canine study. Cystoscopic examination available at 12 to 27 months post PUL displayed normal-appearing mucosa in the prostatic urethra with no evidence of inflammation or encrustation with urethral end-pieces invaginated into the prostate lobes [9] (Fig. 4). Pathological assessment of resected explants at 13, 15, 27, and 43 months further exhibited no evidence of inflammation or foreign body type reaction. Proliferative changes such as neoplasia or hyperplasia were not observed in the urothelium or prostate parenchyma (Fig. 5), and there were no signs of infection.

### Long-term biological mechanism of action

**Fig. 6:**
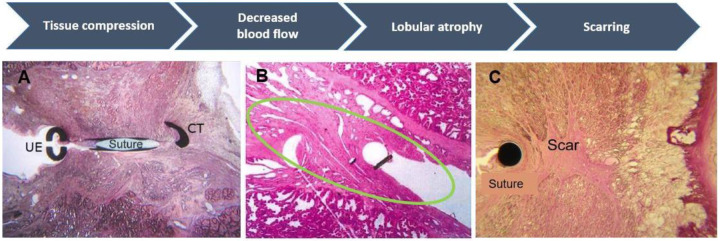
Histological sections of canine prostate showing events leading to tissue remodeling in the zone surrounding the UroLift implant. **A** Localized compression in the implant zone between the Capsular tab (CT) and Urethral End-Piece (UE) results in reduced tissue perfusion. **B** Moderate lobular atrophy and minimal-mild chronic inflammation in tissue surrounding the implant observed at one and six months (the implant circled in green). **C** Stable end-stage healing response characterized by lobular atrophy and scarring in tissue surrounding the implant at 12 months.

Microscopic examination of canine prostate sections revealed the UroLift implant initiates a process of tissue remodeling induced by the localized compression between the capsular tab and urethral end piece (Fig. 5A). Compression likely leads to local ischemia that induces observed tissue atrophy. Within 1 to 6 months post treatment, histologic changes in the compression zone between urethra and capsule are all consistent with a stable tissue remodeling response following implantation and characterized by moderate subacute lobular inflammation, moderate lobular atrophy, and localized glandular duct dilation resulting from compressed prostatic ducts. Increased lobular atrophy and progression to fibrosis and scaring occur at 12 months post implantation (Fig. 5A), with 4.2 mm of scarring observed through most of the atrophic region (Fig. 5A & B). The implant may become fully encapsulated within the prostatic tissue as early as 6 months. Dilation of glandular ducts within the compression zone was also seen in human prostate tissue samples harvested 13 months after treatment with the UroLift System (Fig. 5B). Collectively these results support a long-term biological mechanism of action whereby compression-induced localized tissue remodeling is initiated by implant placement, which then leads to roughly 4 mm of lobular atrophy surrounding the encapsulated fixed implant (Fig. 6). In this way, the tissue fully remodels to the reduced prostate lobe width created initially by the implant alone.

### Implant stability

Review of post-market surveillance provided by the manufacturer from 779,844 UroLift System devices used in the clinic further reveals the stability of the implant as demonstrated by the absence of evidence of migration after a proper deployment. Voluntary reporting yielded a 0.004% (28/779,844) breakage rate and a 0.006% (43/779,844) rate of stone formation from encrustation. Nearly all stones that formed were located in the bladder on the exposed part of the implant and were attributed to improper placement.

## Discussion

Over 200,000 patients worldwide have been treated with PUL conducted with the UroLift System, the first implantable medical device to demonstrate safe and effective improvement of LUTS/BPH without the complications associated with permanent prostatic stents. In numerous clinical studies, PUL has been shown to be minimally invasive, often delivered in the office setting with local anesthesia. Post-operative urinary catheterization is considerably lower than other leading BPH procedures; adverse effects are typically mild to moderate resolving within two to four weeks of treatment; and PUL is the only BPH procedure that has not been associated with new onset sustained erectile or ejaculatory dysfunction [10, 13, 14, 19, 20]. As observed cystoscopically and with other imaging modalities, the UroLift implant pulls the prostatic urethra toward the capsule thereby mechanically reducing obstruction in the prostatic urethra. This is a fundamentally distinct mode of action compared with thermal ablation techniques that induce tissue damage and result in extended periods of edema, retention/catheterization, irritative symptoms and higher risks of urinary infections [21–23]. By preserving the bladder neck and the peri-colliculus tissue around the verumontanum, PUL uniquely preserves ejaculatory function. Resection, vaporization and thermal ablation are all associated with ejaculatory dysfunction due to thermal injury and/or surgical cutting of the internal sphincter and prostatic tissue [24–26]. Erectile dysfunction resulting from cavitating/ablative procedures [27] may likely be due to dispersed energy affecting the nervi erigentes (pelvic splanchnic nerves), which innervate the prostate and sexual organs. Mechanical devices such as PUL avoid this region and this risk [28].

While the acute mechanical nature by which PUL functions has been widely known, this is the first study to elucidate the chronic response of the prostate tissue to the implant, offering a long-term biological understanding of the demonstrated durability of effect. The surgical retreatment rate for PUL is 13.6% at five years post treatment [10], which is comparable to standard BPH procedures [29–31]. Histology shows that the compressive effect of the implant leads to permanent tissue remodeling into the compressed geometry with encapsulation of the implant. The atrophic zone created by the implant is less cellular than the surrounding hyperplastic tissue and consequently may demonstrate less hyperplasia. A series of implants delivered in PUL can create a durable channel while the remainder of the prostate is free to continue its hyperplastic process. One could theorize that this channel could function by allowing bladder pressure to enter the prostate unobstructed further compressing the lobes and thereby dilating the urethra during micturition. Without such a channel, bladder pressure may simply bear down on the obstructed prostate and not open the prostatic urethra sufficiently for flow.

Prostatic stents (introduced in the 1980s) were fraught with complications including migration, encrustation, recurrent urinary tract infections, and tissue overgrowth near the implant, which required a secondary removal procedure [7, 8, 32]. These issues were likely associated with the fact that stents were originally developed for cylindrical vascular anatomy, rather than the confluence of lobes that define the prostatic urethra. The high density of foreign material and the poor fit to the anatomy led to encrustation due to foreign material exposed to standing urine and implant migration either toward the bladder, external sphincter or into the prostatic parenchyma. The UroLift implant departs from this approach as the urologist is able to compress the obstructive prostatic lobe with the delivery device and tailor the suture to reflect the patient’s uniqure prostate anatomy. As such, minimal foreign material is embedded into the prostatic tissue. Furthermore, because each implant is directed toward a focal effect on the prostate lobe, there is no excess material and the urethral component and surrounding urethral tissue invaginate into the compliant transitional zone tissue. Encrustation is very rare when the implant is properly deployed in the prostate. Early reports showed misdeployment into the bladder vesicle as high as 9% [10], but a recent post market study of over 1,400 patients showed this rate to be <1% [12]. The lack of evidence of UroLift implant migration in over 750,000 deployed clinically is likely because the implant is effectively a tissue anchor deployed across a single organ, approximating urethra to capsule. There is no residual or suspending force that would cause the implant to migrate in one direction or the other. The implant reshapes the prostate lobe, either lateral or middle, to a compressed configuration. Histology shows that the surrounding tissue then remodels into this new shape and in some instances encapsulates the implant. Upon completion of remodeling, it can be hypothesized that the compression initiated by the suture diminishes and the newly remodeled tissue contributes to the long-term effect. The total extent of atrophy produced by each UroLift implant in this canine model is ~4 mm; the atrophic tissue may act as a histologic scar, which reinforces the open configuration of the lobe. These cellular changes reflect the expected response of tissue following implantation, are indicative of a stable healing process, and may contribute to the durability of the treatment. One may extrapolate that the human prostate would have a similar remodeling and atrophic response following treatment.

The UroLift implant is made of chemically and biologically inactive materials, commonly used in other implants, thus minimizing the risk of unfavorable interactions between the implant and host tissue [17]. Evaluation from human and canine tissues containing the implant components reveal no evidence of significant inflammation at the tissue-implant interface and in tissues in proximity to the implant at any timepoint post-implantation. The inflammatory response was categorized as a minimal-mild chronic inflammation and minimal-mild fibrosis and was considered a stable end-stage healing response that is typical for a biocompatible permanent device. In addition, scanning electron microscopy examination of UroLift explants after 15 months of implantation in canine showed durability of the materials with no signs of corrosion degradation such as material discoloration, or rough surfaces in any of the implant parts, further indicating that the implants were biocompatible and chemically stable.

A key strength of this study is the strong visual evidence of both the short-term mechanical and long-term biological mechanisms of action that we present utilizing canine and human explant tissue. The evaluation of the animals at different time points assists in the understanding of the tissue remodeling process. This study includes data that is rarely analyzed in peer-reviewed literature, namely adverse event and complaint reporting chronicled by the manufacturer. Some limitations to consider in this study include the small sample sizes of tissue samples. Minimal animal experimentation was conducted to adequately assess implant biocompatibility, and due to the low retreatment rate of PUL, few resection samples were available after treatment.

In summary, numerous clinical studies have demonstrated the prostatic urethral lift procedure as a safe, effective, and durable minimally invasive treatment for BPH. By not relying on tissue damage and recovery to reduce prostatic obstruction, this mechanical approach avoids extensive post operative retention, irritation, and infection that can be associated with thermal ablation and standard surgery. In addition to the acute reshaping of the prostatic urethra induced by the implants, the prostatic tissue remodels to assist in long term durability. The implant causes localized compression that induces chronic remodeling, scarring and fibrosis, reducing the effect of continued prostatic hyperplasia in the area of implantation. When placed properly, low rates of implant removals are expected as the implant safely affects cellular changes with minimal risk of migration, encrustation, or breakage.
