# Correction: The UroLift implant: mechanism behind rapid and durable relief from prostatic obstruction

**DOI:** 10.1038/s41391-021-00457-7

**Published:** 2022-01-12

**Authors:** Claus G. Roehrborn, Peter T. Chin, Henry H. Woo

**Affiliations:** 1 The University of Texas Southwestern Medical Center, Dallas, TX USA; 2 School of Medicine, University of Wollongong & SouthCoast Urology, Wollongong, NSW Australia; 3 College of Health and Medicine, Australian National University & The University of Sydney & SAN Prostate Centre of Excellence, Sydney Adventist Hospital, Wahroonga, NSW Australia

**Keywords:** Prostatic diseases, Translational research

Correction to: *Prostate Cancer Prostatic Diseases* 10.1038/s41391-021-00434-0, published online 06 August 2021

The article “The UroLift implant: mechanism behind rapid and durable relief from prostatic obstruction”, written by Claus G. Roehrborn, Peter T. Chin & Henry H. Woo, was originally published electronically on the publisher’s internet portal on 6 August 2021 without open access. With the author(s)’ decision to opt for Open Choice the copyright of the article changed on 8 September 2021 to ©. The Author(s) 2021 and the article is forthwith distributed under a Creative Commons Attribution 4.0 International License, which permits use, sharing, adaptation, distribution and reproduction in any medium or format, as long as you give appropriate credit to the original author(s) and the source, provide a link to the Creative Commons licence, and indicate if changes were made. The images or other third party material in this article are included in the article’s Creative Commons licence, unless indicated otherwise in a credit line to the material. If material is not included in the article’s Creative Commons licence and your intended use is not permitted by statutory regulation or exceeds the permitted use, you will need to obtain permission directly from the copyright holder. To view a copy of this licence, visit http://creativecommons.org/licenses/by/4.0.

